# Supplementary figures and images for: Forecasting Bitcoin closing price series using linear regression and neural networks models
Source: PeerJ Comput Sci. 2020 Jul 6;6:e279. doi: 10.7717/peerj-cs.279 (PMC7924725; doi:10.7717/peerj-cs.279)

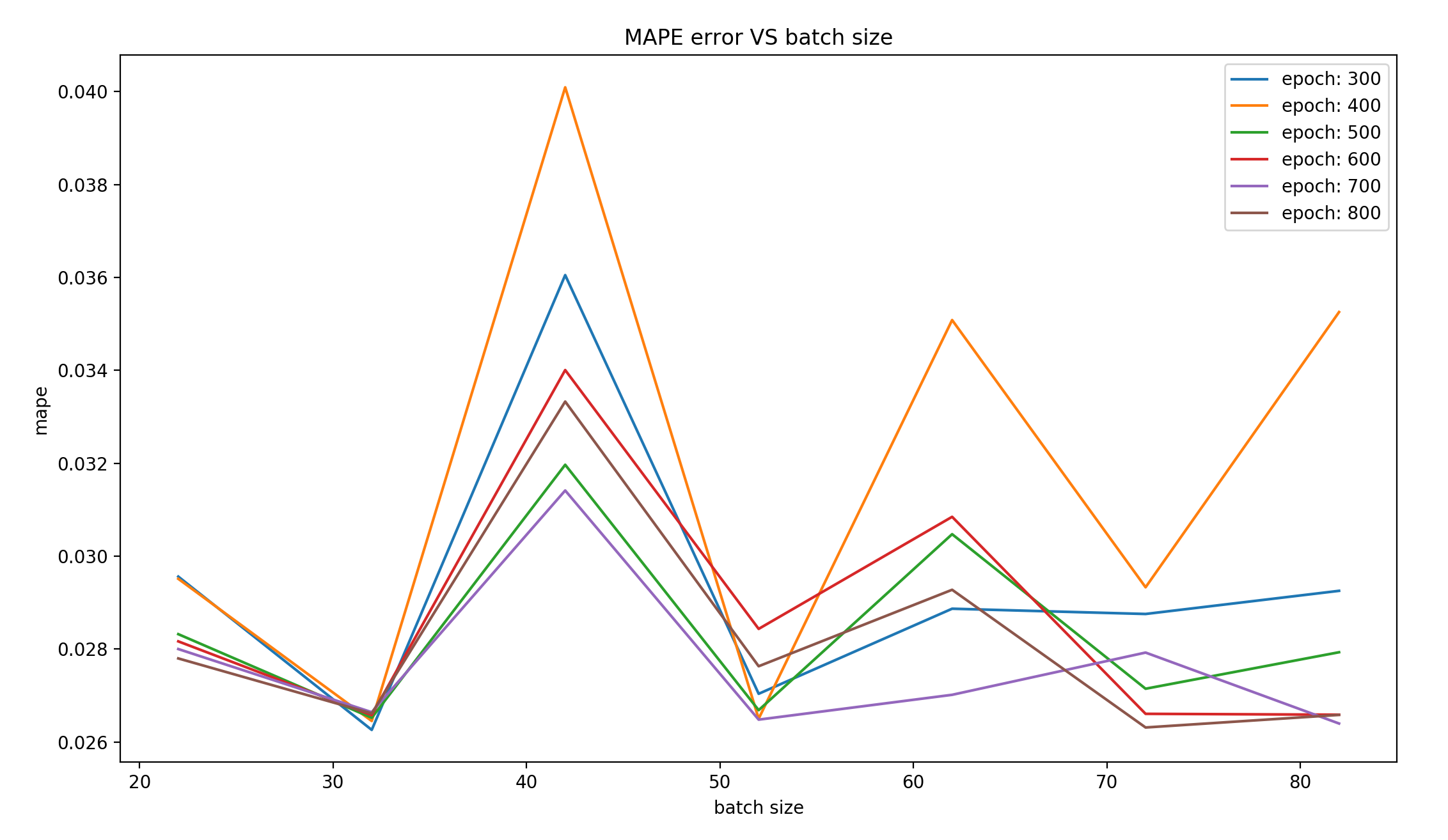

Supplement: Supplemental Information 1 — The project aims to forecast both cryptocurrency and traditional stock market price series using different approaches, such as linear regression models and neural networks. [file peerj-cs-06-279-s001.zip › fbp-master/hyperparameters_check/hyperparameters_check_mape.png]

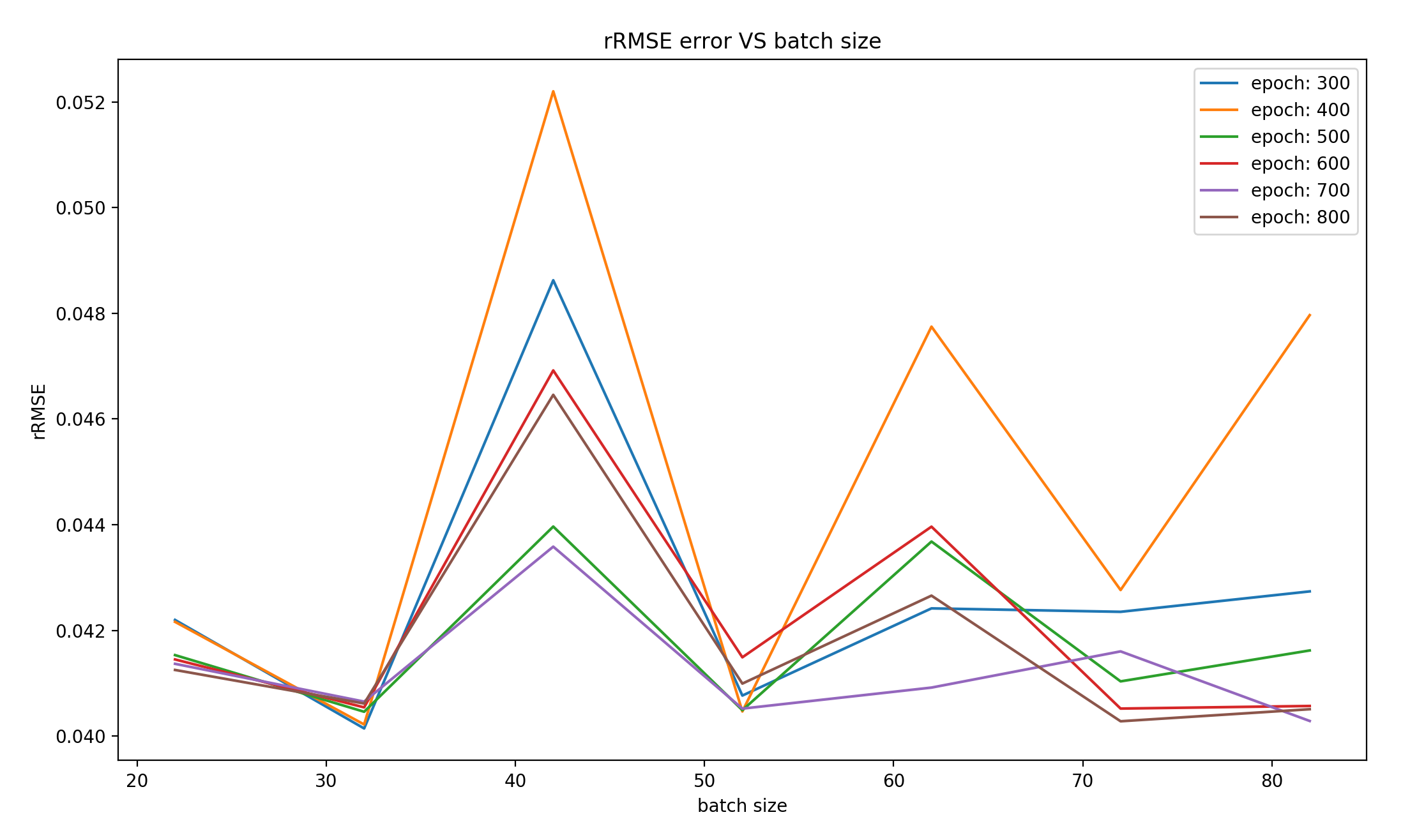

Supplement: Supplemental Information 1 — The project aims to forecast both cryptocurrency and traditional stock market price series using different approaches, such as linear regression models and neural networks. [file peerj-cs-06-279-s001.zip › fbp-master/hyperparameters_check/hyperparameters_check_rRMSE.png]
